# The odd man out in Sub-Saharan Africa: understanding the tobacco use prevalence in Madagascar

**DOI:** 10.1186/1471-2458-13-856

**Published:** 2013-09-17

**Authors:** Hadii M Mamudu, Rijo M John, Sreenivas P Veeranki, Ahmed E Ogwell Ouma

**Affiliations:** 1 Department of Health Services Management and Policy, College of Public Health, East Tennessee State University, Johnson City, TN, USA; 2 Indian Institute of Technology, Jodhpur, Rajasthan, India; 3 Department of Pediatrics, Division of General Pediatrics, Vanderbilt University School of Medicine, Nashville, TN, USA; 4 Tobacco Control Division, WHO Regional Office for Africa, Brazzaville, Congo

**Keywords:** Madagascar, Tobacco use, Tobacco control, Tobacco industry, Sub-Saharan Africa

## Abstract

**Background:**

The tobacco industry has globalized and tobacco use continues to increase in low- and middle-income countries. Yet, the data and research to inform policy initiatives for addressing this phenomenon is sparse. This study aims to estimate the prevalence of adult tobacco use in 17 Sub-Saharan Africa (SSA) countries, and to identify key factors associated with adult tobacco consumption choices (smoked, smokeless tobacco and dual use) in Madagascar.

**Methods:**

We used Demographic Health Survey for estimating tobacco use prevalence among adults in SSA. A multinomial logistic regression model was used to identify key determinants of adult tobacco consumption choices in Madagascar.

**Results:**

While differences in tobacco use exist in SSA, Madagascar has exceptionally higher prevalence rates (48.9% of males; 10.3% of females). The regression analyses showed complexity of tobacco use in Madagascar and identified age, education, wealth, employment, marriage, religion and place of residence as factors significantly associated with the choice of tobacco use among males, while age, wealth, and employment were significantly associated with that of females. The effects, however, differ across the three choices of tobacco use compared to non-use.

**Conclusions:**

Tobacco use in Madagascar was higher than the other 16 SSA countries. Although the government continues to enact policies to address the problem, there is a need for effective implementation and enforcement. There is also the need for health education to modify social norms and denormalize tobacco use.

## Background

Tobacco use in many Sub-Saharan Africa (SSA) countries is generally low [1], but like many low- and middle-income countries (LMICs) around the world, the volume of cigarette consumption has been increasing over the past decades [2,3]. While there are several determinants for this increasing trend of tobacco use in LMICs, a key contributory factor has been the aggressive market penetration by Transnational Tobacco Companies (TTCs) [4-6]. Understanding tobacco use and the role of TTCs in exacerbating the problem has, however, been hampered by paucity of data and sparse research [7]. This development has generated the need for more investigations, and policy and programmatic interventions to combat tobacco use in LMICs such as those in SSA.

In 2003, the World Health Organization (WHO) Framework Convention on Tobacco Control (FCTC), the first international public health treaty negotiated under the auspices of the WHO [8], was developed to deal with the globalization of tobacco use, and has been ratified by most SSA countries [9]. Still, the generally low, but increasing rates of tobacco use in SSA countries [3,10], along with demographic and socioeconomic changes such as increasing incomes and urbanization [2], and relatively weak tobacco control policies and programs [1] provide a potential market for the tobacco industry to exploit. Although research to provide insight into adult and youth tobacco use in SSA [11-16] has slowly been growing over the past decade, there is an inadequate analysis across countries and limited information on many countries, including Madagascar [17]. Therefore, this study investigates adult tobacco use in SSA with a greater emphasis on Madagascar because of high rates of prevalence of tobacco use compared with SSA countries. Additionally, Madagascar requires more consideration because it is not only one of the few LMICs and SSA countries with scarce information on adult tobacco use [1] but also faces a bleak public health future as over two-thirds of the population live below the poverty line [18], its Human Development Index ranking is one of the lowest in the world [19], and tobacco-induced non-communicable diseases (NCDs) such as cardiovascular diseases, cancer and chronic obstructive pulmonary disease have emerged as major causes of mortality [18].

Madagascar is the largest island in the Indian Ocean and the 4th largest island in the world with a population of about 21 million in 2010 [18]. The presence of the tobacco industry in the country dates back to the 1950s when the Tobaccor Group from France established a cigarette manufacturing and distribution network [5]. In 2001, Imperial Tobacco Limited acquired the Tobaccor Group and became a near monopoly in Madagascar with easier access to tobacco markets in Eastern and Southern Africa [5,20,21]. Simultaneously, to combat the increasing tobacco use in the country, the government has taken progressive steps since 1998 to enact policies such as taxes on tobacco products (76% of retail price [22]), health warnings (in French and Malagasy) on tobacco packages (including the graphic warnings), ban on the use of descriptors and tobacco products in certain venues (e.g. workplaces, public transport and public places), and ban on tobacco advertising, promotion and sponsorship, ban on the sale of cigarettes to and by minors (<18 years) with point of sale notice, ban on cigarette vending machines, and prohibition of illicit tobacco trade (manufacturing and counterfeiting) [23]. The co-existence of a strong tobacco industry working to bolster consumption through marketing, promotions and corporate social responsibility activities, and progressive tobacco control efforts by the government generates the need to identify where to target the limited resources to reduce tobacco use prevalence in the population.

The objective of this study was to estimate adult tobacco use prevalence in 17 SSA countries with a greater focus on understanding tobacco use in Madagascar. Along with estimating prevalence of adult tobacco use in these 17 countries, we will further assess the prevalence, and identify key determinants of choice of various forms of tobacco use (smoked, smokeless and dual use) among adults aged 15–49 years in Madagascar. We hypothesize that there is a wide variation in tobacco use in SSA countries, and that tobacco use in Madagascar is associated with several socioeconomic and contextual factors. The study provides an overview of tobacco use in SSA, and facilitates a comprehensive understanding of the situation in Madagascar to inform policy and programmatic initiatives to control the problem as well as support policies that ensure the implementation of the FCTC.

## Methods

Initially, we conducted a preliminary analysis to estimate tobacco use prevalence among both adult male and female residents in 17 SSA countries using the Demographic Health Survey (DHS), 2005–2010. The DHS was a stratified, two-stage cluster sampling design, where occupied households (e.g., 18,083 for Madagascar in 2008) were given a general household questionnaire and in half of these households, additional questionnaires were administered for the males’ interview. Detailed description of the survey, including methodology, questionnaire development, administration, collection of information and management of data by survey weights to make it representative of the respective populations have been discussed in earlier studies and reports [11,24], and are also available online at ICF International [25].

Subsequently, we conducted a comprehensive analysis to understand tobacco use among adults in Madagascar. As stated earlier, Madagascar was chosen because the tobacco use among both adult males and females was much higher than those in the other 16 SSA countries. The overall response rate for the household survey in Madagascar was 98.8%. We examined and compared the choice behavior of males and females aged 15–49 years with respect to their tobacco consumption to ensure uniformity of data in the regression analyses. This study, which is analyses of the DHS data, was approved by the Institutional Review Board of East Tennessee State University.

The participants in the DHS were asked several health-related questions, including their tobacco use behavior. The choice behavior of a participant’s tobacco consumption was determined by positive self-reported responses to whether s/he currently smoked cigarettes or smoked/used any type of tobacco and the type of tobacco s/he currently smoked/used (Pipe/Chewing tobacco/ Snuff/Other) [26,27]. Thus, a typical participant in the survey was faced with a choice between smoked and smokeless tobacco products (SLTs). Therefore, a participant can decide which of the four tobacco use categories applies to her or him: no consumption of any tobacco products, consumption of only smoked tobacco products, consumption of only SLTs, or consumption of both smoked tobacco products and SLTs (dual use). Approximately 96% of females aged 15–49 years (n=17,375) and 93% of males aged 15–59 years (n=8,586) provided information on their current use of tobacco products, which include smoked and smokeless tobacco products.

Multinomial Logit Model (MNLM), which simultaneously estimates binary logits for all possible comparisons among the outcome categories, is well suited to examine such multiple outcomes [28]. We specified each nominal outcome as a nonlinear function of the independent variables. The MNLM can be formally stated as follows. Let *y* be a dependent variable with *J* nominal outcomes. The *J* categories are numbered 1 through J, but are not ordered in any way. Let Pr(*y*_*i*_ = *m* | ***X***_i_) be the probability of observing outcome *m* for individual *i* given ***X***, the set of explanatory variables. As a probability model, the MNLM can then be written as:

$$\ln\;\Omega_{m|b\;}\left(X\right)=\ln\frac{\mathrm{Pr}\left(y=m|X\right)}{\mathrm{Pr}\left(y=b|X\right)}=X\beta_{{}_{m|b}}\mathrm{for}\;m=1\;\mathrm{to}\;J$$

where *b* is the base category, also referred to as the comparison group. These *J* equations can be solved to compute the predicted probabilities:

$$\mathrm{Pr}\left(y=m|X\right)=\frac{\exp\left(X\beta_{m|b}\right)}{\Sigma_{j=1}^{J}\exp\left(X\beta_{j|b}\right)}$$

In this study, the dependent variable (y) has 4 outcome categories (*J*) namely, no tobacco use (hereafter “none”) (*b*), using only smoked tobacco products (hereafter “smoked”), using only smokeless tobacco products (hereafter “SLTs”), and using both smoked tobacco products and SLTs products (hereafter “dual use”). The independent variables (*X*) included gender, age in years, education status (no education, primary education, high-school education, or university education), standardized wealth index^a^ used as a proxy measure for income that was not collected in the survey, occupation status (unemployed, agricultural labors, service manual labors, or service non-manual labors), marital status (married or unmarried), religious beliefs (Christianity, Islam, Traditional religions, or other religions) and place of residence (rural or urban). Although the choice of these variables was informed by the literature [11,29,30], it was simultaneously constrained by the information available in the DHS dataset [25]. All the variables, except age and wealth index, were categorical. Although price is an important determinant of tobacco use [6], it was not included in the regression because the price paid for tobacco was not collected by the survey and the cross-sectional nature of data limited variation in tobacco prices across time.

Based on the established gender gradient in tobacco use in SSA [1,10], the analyses were done separately for males and females. Descriptive statistics of three types of tobacco use (smoked, SLTs, and dual use) were reported for males and females using proportions. However, MNLM was not reported for dual use among females due to insufficient sample size (n=18). Additionally, in order to make comparisons more meaningful, data for a uniform age category (15–49 years) were used for both males and females to estimate the MNLM. The MNLM models were checked for possible multicollinearity, but it did not warrant dropping any variables. Robust standard errors were used to improve estimates because of lack of information on structure of heteroskedasticity of the data. The Chi square goodness of fit statistics was estimated to check the overall fit of the model and is reported along with the MNLM results. All models were estimated using probability weights from the survey and Stata 11.0 software (College Station, Texas, USA).

## Results

**Figure 1:**
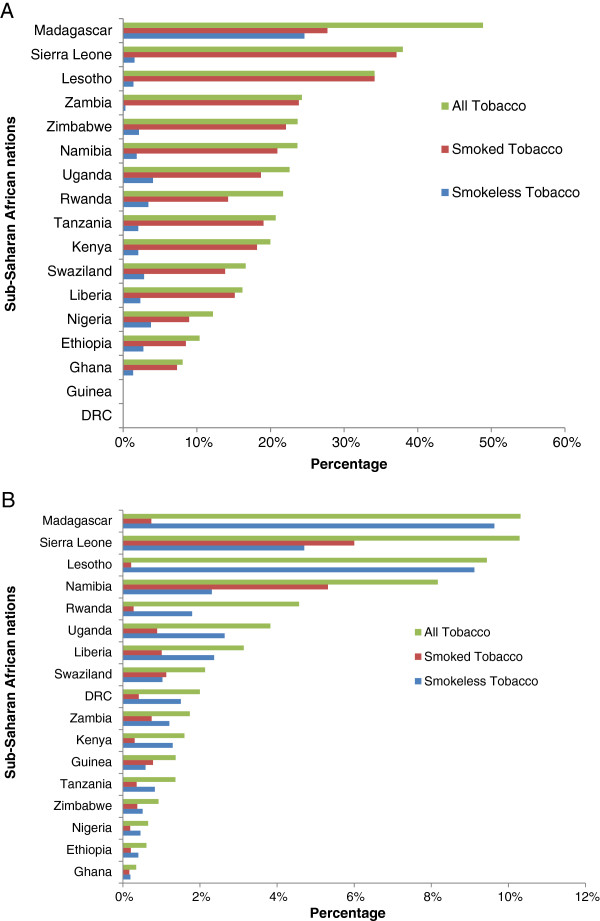
**Tobacco use among adults in Sub-Saharan African Countries.A**. Tobacco use among adult males in Sub- Saharan African countries, Demographic Health Survey, 2005-2010. **B**. Tobacco use among adult females in Sub- Saharan African countries, Demographic Health Survey, 2005-2010.

The results of the preliminary analysis of adult tobacco use in the 17 SSA countries are presented in Figures 1A and 1B. The Figure 1A shows that for males aged 15–59 years, the prevalence of smoking ranged from 7.31% in Ghana to 37.11% in Sierra Leone, SLTs use ranged from 0.29% in Zambia to 24.62% in Madagascar, and use of all tobacco products ranged from 8.08% in Ghana to 48.87% in Madagascar. Figure 1B shows that among females aged 15–49 years, the prevalence of smoking ranged from 0.17% in Ghana to 6.0% in Sierra Leone, SLTs use ranged from 0.20% in Ghana to 9.64% in Madagascar, and use of all tobacco products ranged from 0.34% in Ghana to 10.29% in Madagascar. These results demonstrated that the prevalence of tobacco use in Madagascar was much higher than other 16 SSA countries in the DHS dataset, indicating the need for comprehensive investigation into tobacco use in this country.

Table 1 presents the prevalence of tobacco use in Madagascar by gender for all the three types of tobacco use, i.e., smoked (28.5% males, 0.77% females), SLTs use (24.6% males, 9.6% females) and dual use (4.3 males, 0.1% females). The prevalence of tobacco use has a strong gender gradient with 48.9% of males currently using tobacco in some form, while only 10.3% of women do so (p <0.05).

**Table 1:** Prevalence of tobacco use in Madagascar among females aged 15–49 (n= 17,375) and males aged 15–59 years (n=8,586), Demographic Health Survey, 2008-09

**Characteristics**	**Smoked tobacco products**		**Smokeless tobacco products**		**Dual use of tobacco products**		**All tobacco**^**#**^	
	**Female (%)**	**Male (%)**	**Female (%)**	**Male (%)**	**Female (%)**	**Male (%)**	**Female (%)**	**Male (%)**
**N**	118	2152	1579	1712	18	359	1715	4223
**Total**	0.77	28.50	9.60	24.60	0.09	4.30	10.3	48.90
**Age (in years)**								
15-19	0.60	14.87	3.4	8.40	0.15	1.13	3.82	22.13
20-24	0.43	35.82	7.4	17.14	0.00	4.57	7.82	48.39
25-29	0.60	33.61	9.7	24.35	0.07	5.34	10.25	52.62
30-34	0.73	31.38	12.0	27.94	0.06	4.40	12.68	54.92
35-39	0.95	32.36	13.8	30.65	0.24	5.11	14.52	57.90
40-44	1.17	29.91	14.2	32.18	0.06	6.45	15.34	55.64
45-49	1.53	32.88	15.1	35.79	0.04	6.33	16.58	62.34
50-54		25.71		40.46		3.69		62.49
55-59		24.42		41.86		4.33		61.95
**Education**								
No Education	0.83	32.37	15.2	33.47	0.29	5.22	15.74	60.62
Primary	0.38	27.27	11.7	31.73	0.05	4.83	11.98	54.17
High-school	1.27	28.37	3.6	10.51	0.04	3.17	4.85	35.71
University	2.06	29.57	0.0	2.70	0.00	1.37	2.06	30.90
**Wealth Index (in quintiles)**								
Lowest	0.38	30.07	12.9	34.36	0.12	4.67	13.18	59.77
Second	0.57	27.04	13.5	31.19	0.11	4.04	13.94	54.18
Middle	0.42	25.20	11.6	32.83	0.10	4.99	11.89	53.04
Fourth	0.43	28.95	8.7	22.59	0.00	5.76	9.09	45.78
Highest	1.74	30.74	3.7	8.09	0.13	2.25	5.36	36.59
**Occupation**								
Unemployed	1.09	11.56	2.5	2.31	0.01	0.51	3.59	13.36
Agriculture	0.34	29.04	12.5	32.16	0.09	5.23	12.77	55.97
Service-manual	1.70	35.37	7.9	15.06	0.17	3.20	9.44	47.24
Service-non-manual	1.77	32.50	1.4	8.80	0.13	3.67	3.02	37.64
**Marital status**								
Unmarried	0.75	25.13	7.84	16.26	0.08	2.82	8.51	38.58
Married	0.78	31.04	10.81	30.96	0.10	5.37	11.49	56.63
**Religion**								
Other Religion*	0.56	32.48	9.7	23.19	0.15	3.95	10.11	51.72
Christian	0.84	26.45	9.7	25.42	0.08	4.41	10.46	47.46
Muslim	0.00	39.69	1.7	14.91	0.00	9.45	1.65	45.14
Traditional	0.96	35.05	9.7	22.75	0.00	1.84	10.67	55.96
**Residence**								
Urban	1.69	33.78	4.4	8.05	0.18	2.75	5.92	39.08
Rural	0.58	27.48	10.7	27.81	0.08	4.56	11.23	50.72

* All other religious groups which are not Christian, not Muslim or not traditional.

^#^ Prevalence for smoked and smokeless tobacco use is not mutually exclusive. Therefore, the totals in the last two columns are summations of the corresponding smoked and SLT percentages minus dual use.

Among males, SLTs use prevalence increased with age, but smoked and dual use of tobacco products peaked during 20–24 years (35.82%) and 40–44 years (6.45%), respectively. It can also be observed that the prevalence of the use of all forms of tobacco products was higher for males with no or lower than primary education. Additionally, while males employed in agriculture had higher prevalence of SLTs use (32.16%) and dual use (5.23%), those in service-manual employment had high prevalence of smoking (35.37%) than others. The prevalence of all forms of tobacco products was more among married (56.63%) than unmarried males (38.58%). While Christian males had the highest prevalence of SLTs use (25.42%), the prevalence of smoking (39.69%) or dual use (9.45%) was highest among Muslims males. While the prevalence of SLTs use (27.81%) and dual use (4.56%) was higher among males residing in rural areas, those residing in urban areas had higher prevalence of smoking (33.78%).

Among females, the prevalence of dual use of tobacco products is much less (0.09%) compared to that of smoking (9.60%) and SLTs use (0.77%). While the prevalence of SLTs use and smoking increased with age, that of dual use peaked during 35–39 years. Although the highest prevalence of SLTs and dual use was among females with no education, the highest smoking prevalence was among those with high-school education and above. With increase in wealth, the prevalence of SLTs use decreased, but vice versa with that of smoking and dual use. Furthermore, similar to males, female agriculture employees had the highest prevalence of SLTs use (12.5%), however, those in non-manual employment had the highest smoking prevalence (1.77%). The prevalence of the use of all forms of tobacco products was higher among married females and Muslim females had the least prevalence of the use of any tobacco product. Finally, females residing in rural areas had higher prevalence of SLTs use (10.7%), while those in urban areas had higher smoking prevalence (1.69%).

A multinomial logit regression model was initially estimated for males and females combined and it revealed that the relative risks of being a smoker, SLT user, or dual user of tobacco were 66 times [RRR = 65.72; 95% CI (51.49, 83.89)], 4 times [RRR = 3.94, 95% CI (3.550, 4.379)], and 90 times [RRR = 90.29; 95% CI (49.48, 164.8)] higher for males compared to females, respectively. However, considering the strong gender gradient in tobacco use prevalence (Table 1) and higher relative risks of tobacco use among males than females (as stated earlier), regression models were conducted separately for males and females. (The results of the combined analysis can be made available upon request.)

Table 2 presents the relative risk ratios (RRRs) from the multinomial logit regression for the choice of tobacco use among males and females aged 15–49 years. The education and religion variables were dropped from the female regression because the sample was not sufficient for females belonging to certain categories of education and religion in the examination of the various tobacco product choices.

**Table 2:** Key determinants of adult tobacco use in Madagascar, Demographic Health Survey, 2008–09 (Base outcome category: No tobacco use)

	**Smoked tobacco products (Smoking)**				**Smokeless tobacco products (SLTs use)**				**Dual use**	
	**Male**		**Female**		**Male**		**Female**		**Male**	
	**RRR**	**95% CI**	**RRR**	**95% CI**	**RRR**	**95% CI**	**RRR**	**95% CI**	**RRR**	**95% CI**
**Age**										
Age 15-49	1.02***	[1.01,1.03]	1.06***	[1.03,1.08]	1.06***	[1.05,1.07]	1.05***	[1.04,1.05]	1.04***	[1.02,1.06]
**Education (Base: University education)**										
No education	1.39	[0.86,2.25]			7.68*	[1.41,42.00]			3.16	[0.67,14.93]
Primary	1.16	[0.75,1.82]			6.52*	[1.21,35.20]			2.26	[0.50,10.28]
High-School	1.04	[0.69,1.57]			2.49	[0.46,13.50]			1.54	[0.38,6.21]
**Wealth**										
Wealth Index	1.02	[0.89,1.16]	1.58*	[1.11,2.24]	0.45***	[0.38,0.54]	0.59***	[0.52,0.66]	0.70*	[0.51,0.98]
**Occupation (Base: Unemployed)**										
Agriculture	3.41***	[2.46,4.72]	0.32**	[0.14,0.73]	5.26***	[2.74,10.09]	2.36***	[1.76,3.16]	6.58***	[2.32,18.67]
Service-manual	3.63***	[2.62,5.04]	1.03	[0.55,1.92]	5.55***	[2.79,11.03]	2.44***	[1.77,3.37]	4.94**	[1.66,14.69]
Service-non-Manual	2.75***	[1.76,4.30]	0.62	[0.24,1.59]	3.77**	[1.54,9.26]	0.42*	[0.18,1.00]	5.99**	[1.55,23.20]
Married (Base: Unmarried)	1.02	[0.86,1.21]	0.86	[0.54,1.38]	1.22*	[1.01,1.48]	0.96	[0.83,1.11]	1.46*	[1.04,2.06]
**Religion (Base: Christian)**										
Other Religion	1.28**	[1.09,1.51]			0.51***	[0.42,0.62]			0.63**	[0.46,0.86]
Muslim	1.36	[0.77,2.42]			0.11***	[0.03,0.41]			2.14	[0.74,6.21]
Traditional	1.57	[0.96,2.58]			0.52*	[0.29,0.92]			0.29*	[0.09,0.86]
**Residence (Base: Rural)**										
Urban	1.37*	[1.07,1.75]	0.86	[0.45,1.64]	0.81	[0.56,1.16]	1	[0.76,1.31]	1.25	[0.74,2.11]

Notes: RRR - Relative Risk Ratios; 95% confidence intervals in parentheses; Chi-square goodness of fit statistics 829.14 for male regression and 620.28 for females were both significant at p<0.001; Sample size was 7653 for male regression & 17369 for female regression; Age and wealth index are continuous variables and all others are categorical; Analyses were not conducted for dual use among females because of the small n.

* p<0.05, ** p<0.01, *** p<0.001.

Among males, the prevalence of all forms of tobacco use had a positive association with age, i.e., as age increased, the relative risk of use of any tobacco product increased as well. Compared to males with university education, those with no or primary education had 7.7 [RRR = 7.68, 95% CI (1.41, 42.00)] and 6.5 [RRR = 6.52 95% CI (1.21, 35.20)] times higher relative risks of SLTs use. While the wealth index did not have an effect on the choice of male smoking, an increase in a person’s wealth significantly decreased the relative risks of being a SLTs or dual user of tobacco products by a factor of 0.45 [RRR = 0.45, 95% CI (0.38, 0.54)] and 0.7 [RRR = 0.70, 95% CI (0.51, 0.98)] times, respectively. Employed males had higher relative risks of using any form of tobacco products than unemployed. Specifically, males in service-manual employment had higher relative risks of smoking or SLTs use, while those employed in agriculture had higher relative risks of dual use of tobacco products, compared with unemployed males. Married males had 1.22 [RRR = 1.22, 95% CI (1.01, 1.48)] and 1.5 [RRR = 1.46, 95% CI (1.04, 2.06)] times higher relative risk of SLTs and dual use than those unmarried. The relative risks of SLT use were lower among all religious groups compared to Christians. Place of residence had a significant effect only for male smoking and that males residing in urban areas had 1.4 [RRR = 1.37. 95% CI (1.07, 1.75)] times higher relative risk of smoking than those residing in rural areas.

Similar to males, the relative risks of smoking and SLTs use among females increased with age. Wealth had varying effect on the choice of tobacco use among females. While wealth was significantly associated with relative risk of smoking among females [RRR = 1.59, 95% CI (1.11, 2.24)], the risk of SLT use was negative [RRR = 0.59, 95% CI (0.52, 0.66)]. While females in both agriculture and service-manual occupation had higher relative risks of SLT use, those in service-non-manual occupation had lower relative risks compared with unemployed ones. Females in agricultural employment had a lower relative risks for smoking compared to unemployed women. Unlike males, marital status did not affect tobacco consumption choices among females in any significant way. Residing in urban areas did not affect smoking or SLT use behavior among females.

## Discussion

Tobacco use causes several preventable diseases and deaths (5.4 million in 2010) in the world [1], yet, the usage rate has been increasing in LMICs over the past decades and is expected to reach over 80% of the volume of global tobacco consumption by 2030 [3]. As a result, the FCTC was unanimously adopted by the Member States of the World Health Assembly, the governing body of the WHO, in 2003 to curb the problem. As at August 2013, the FCTC has been ratifies by 177 Parties, including Madagascar in September 2004. For many LMICs, however, curbing the increasing trend has been problematic and challenging due to inadequate information on tobacco use. Thus, this study explored tobacco use prevalence in 17 SSA countries with a comprehensive investigation into Madagascar to provide information for policy and programmatic interventions as well as facilitate the implementation of the FCTC. Preliminary comparative analysis of the DHS dataset conducted in these 17 SSA countries showed that tobacco use prevalence, particularly SLTs was much higher in Madagascar (Figures 1A and B). Indeed, nearly half of Madagascan adult males in this study reported that they used some form of tobacco product (48.90%). SLTs use prevalence (24.62%) was at least 20 percentage points higher than any of the other countries. This higher level of SLTs use prevalence in the country may be due to the large proportion of Madagascans with South Asian origin as previous studies have documented that South Asians are highest SLTs users in the world [31-33].

The study provided a comprehensive investigation into adult tobacco use in Madagascar by estimating the prevalence and identifying key factors associated with the risk of tobacco consumption. While the prevalence of use of any form of tobacco products (smoked, SLTs, and dual use) in Madagascar was high, the overall prevalence was particularly high among males (28.5% smoking, 24.6% SLTs use, and 4.3% dual use) in a region of the world (i.e., SSA) noted for generally low tobacco use and the epidemic is yet to take off [1,10]. When this high prevalence rate is coupled with the fact that over two-fifths of all mortality in Madagascar is due to NCDs [18], the situation in the country resembles those in more advanced stages of the Tobacco Epidemic Model, which describes the progression of the tobacco epidemic in countries based on the experience of developed countries [10]. Simultaneously, the gender gap supports the modified Tobacco Epidemic Model because it suggests that males and females in many LMICs have different tobacco epidemic trajectories [10]. This high and increasing level of tobacco use, amidst the government’s efforts to enact and implement some of the effective tobacco control measures in SSA in conformity with the FCTC [5,21,34,35], supports the thesis that the development of effective domestic tobacco control programs requires convergence of institutions, agendas, networks, socioeconomic factors and ideas [9]. In fact, as of May 2013, Madagascar’s excise tax rate of 76% of retail price of tobacco products [22] was not only the highest in SSA but also above the 75% recommended by the WHO [35,36], yet the prevalence of tobacco use was highest among the countries in this study, which demands longitudinal research into causal relationship between price of tobacco products and tobacco use. Nevertheless, with these high rates of tobacco use prevalence and incidence of NCDs in the country, Madagascar faces the double-burden of disease and is now at the stage of tobacco epidemic where there is a necessity for combined aggressive prevention strategies and cessation efforts.

Among adult males and females in Madagascar, although the relative risk of use of tobacco products increased with age, the magnitude of association was very low. This increase in relative risks of tobacco use with increase in age suggests that tobacco use in Madagascar is predominant among adults and necessitates public health education campaigns to inform adults about the health hazards of not only smoking but also SLTs use [1]. Similar to previous studies from other countries [37], we found socioeconomic status (SES) indicators, i.e., education, wealth and occupation, as key determinants of adult tobacco use in Madagascar with varying effects on males and females and on the type of tobacco used. Education particularly had a significant effect on prevalence of SLTs use among males in Madagascar, and those with no or primary education had 7.7 and 6.5 times higher relative risks of being a SLTs user compared with those with university education. While this phenomenon may be attributed to cultural heritage, i.e., a large segment of the population having South Asian origin, the lack of knowledge about the negative health effects of SLTs among people with no or only primary level education in the country may have exacerbated the phenomenon. This suggests the need to increase awareness programs about the ill effects of SLTs, while finding ways to reach the lower educated people with this information or implementing prevention programs tailored for such people.

While increase in wealth index has no effect on smoking among males, it significantly reduced the relative risks of the prevalence of SLTs and dual use among them. This is consistent with research on SLTs from Southeast Asia that considered SLTs as “poor man’s cigarettes” [38]. However, the higher relative risks of smoking prevalence of 1.6 times with increase in wealth index among females merits attention in the country. The tobacco industry target of working and middle class women in LIMICs with Western images and messages of feminine liberation through smoking has been documented [39-41], and it probably contributes to the observed phenomenon among females in Madagascar. Thus, it is important for the government and the public health community to ensure that the tobacco industry in the country does not circumvent the ban on advertising, marketing and promotions due to the potential effects of increasing tobacco use, particularly among wealthy females in the country.

The effects of occupational status on tobacco use showed that usage was generally less among the unemployed, although increases in wealth appears to reduce the relative risks of using SLTs among males and females. These generally higher relative risks of tobacco use among the employed when compared with the unemployed may be due to the issue of affordability. Perhaps, the unemployed did not have the necessary money to purchase tobacco products. It should, however, be noted that compared to unemployed females, the relative risks of prevalence of SLTs use among females in service-non-manual employment was significantly lower. This suggests that the type of employment for females in Madagascar could be an indicator of the type of tobacco they decide to consume. Thus, targeting tobacco prevention and cessation activities at workplaces could be a strategy to address the high prevalence of tobacco use in the country.

Socio-cultural factors, including marital status and religion, have been identified as important factors for understanding tobacco use, especially among women in many LMICs [39]. Although the prevalence of all types of tobacco use was higher among married people, the relative risk was higher and significant only in the case of SLTs and dual use among married males. These results about the effects of marital status of Madagascan males on tobacco use prevalence are not consistent with studies from other parts of SSA where marriage was found to be a protective factor against tobacco use [11], suggesting the complexity of cultural effects on tobacco use in SSA countries. Nevertheless, the significant difference in the rate of dual use between married and unmarried males indicates that a larger percentage of the female population in Madagascar were exposed to secondhand smoke (SHS) because the smoking behavior of a married male is an indicator of SHS exposure of the spouse [42]. With the well-documented evidence on the negative health effects of involuntary or passive smoking by nonsmokers [43,44], these results suggest the need to target married males with such messages because knowledge about negative health effects of SHS is associated with decreased tobacco use and motivation for cessation [1,43,45]. Additionally, it is important for the public health community in the country to advocate for smokefree households to protect nonsmoking married females and children from involuntary or passive smoking.

Other key determinants of tobacco use prevalence in Madagascar included place of residence, which had positive effects only on smoking among males and dual use among females. In this respect, urbanization has long been identified as an important reason for the increasing tobacco use in LMICs over the past decades [2,6]. While SLTs use was more prevalent among rural dwellers than urban dwellers (27.81% vs. 8.05% for males; 10.7% vs. 4.4% for females), smoking was more prevalent among urban than rural dwellers (33.78% vs. 27.48% for males; and 1.69% vs. 0.58% for females). The higher relative risk of prevalence of smoking among males residing in urban areas is consistent with previous studies on adult tobacco use in Africa [46,47]. In fact, the *Tobacco Report Magazine* reported in 2009 that about 50% of all cigarette consumption took place in Antananarivo, the administrative capital and the largest urban area in the country [5]. With the decreasing rural population vis-à-vis the increasing urban population (at a growth rate of 4% in the past decade [18]), this finding should inform public health officials about the impending health epidemic in the country, especially because Imperial Tobacco Limited has been working aggressively since the acquisition of Tobaccor Group in 2001 to bolster tobacco use [5,20,21]. Thus, policymakers in Madagascar should implement and enforce the existing policies in a manner consistent with the FCTC and with engagement in educational and advocacy campaigns that will denormalize tobacco use and the industry. Given the rural–urban divide in the type of tobacco use, while campaigns involving smoking should mostly target urban residents, those involving SLTs use should mostly target rural residents.

### Limitations

This study was cross-sectional in nature, which limited the ability to make any causal inferences. Additionally, the time variation in the preliminary analysis presents an analytical challenge as it does not take into consideration changes in the general tobacco control environment. However, the DHS surveys conducted for 17 SSA countries during different time periods provide the latest comparable information on tobacco use in respective countries. Moreover, age truncation (15–49 years) limited the ability to provide an overall picture of adult tobacco use in the country. Further, the survey responses were based on self-reports, which were subjected to recall bias with no independent means of validation. Another limitation is that although the choice of the explanatory variables for the study was informed by the literature, it was simultaneously constrained by information available in the DHS dataset for Madagascar. As such, other variables such as tobacco industry promotions, smoking and anti-smoking media messages, and receptivity and/or exposure to smoking/tobacco use cessation programs that might potentially influence adult males and females in Madagascar were not included. The different sample sizes for males and females and low sample sizes for some demographic variables in the study resulted in wider confidence intervals, leading to less precision in the interpretation of the regression estimates. Regardless, this study provided the first comprehensive investigation into adult tobacco use in Madagascar that could assist the government and public health community to target their efforts to reduce the high level of tobacco use and thereby reduce the increasing incidence of tobacco-induced NCDs.

## Conclusion

Preliminary analysis of tobacco use in 17 SSA countries identified that tobacco use prevalence in Madagascar was much higher than most SSA countries. This high level of adult tobacco use prevalence occurs amidst increasing incidence of tobacco-induced NCDs in a country where the majority lives on less than one US dollar a day and the government spends less than 4% of its GDP on health services, which raises a major public health concern. This situation suggests that tobacco use in Madagascar should not only be considered as a health and/or economic issue but also a human rights issue, particularly with the Imperial Tobacco Limited’s efforts to exploit the tobacco market in the country. Along with current progress in tobacco control, the government and public health community of Madagascar should engage in health education efforts to make all forms of tobacco use socially unacceptable, while discouraging tobacco use initiation and encouraging cessation. In this respect, the large gender gap in tobacco use prevalence suggests that it is important to educate married couples in the country about the health effects of SHS for nonsmokers and involuntary SHS exposure and policymakers and health professionals should consider integrating the idea of smoke-free household into their health campaigns. Moreover, there should be efforts to denormalize the tobacco industry such that the corporate social responsibility programs of Imperial Tobacco Limited would not cloud the perceptions of the population that the tobacco industry is the “vector” of tobacco use and tobacco-induced diseases such as NCDs [48,49].

## Endnote

^a^Wealth Index used information on household ownership of consumer items, ranging from a television to a bicycle, as well as characteristics of a respondent’s dwelling such as its source of drinking water, sanitation facilities, and type of flooring material used. Each asset was assigned a weight (factor score) generated through principal components analysis, and the resulting asset scores were standardized in relation to a normal distribution with a mean of zero and standard deviation of one. Each household was then assigned a score for each asset, and the scores were summed for each household.

## Competing interests

The authors have no competing interests to declare.

## Author’s contribution

HMM wrote the first draft of the manuscript with input from RMJ, who was involved with data acquisition and analysis. SPV helped with the interpretation of the results and revision of the manuscript. AEOO provided substantive input and revision of the manuscript. All authors contributed to the several revisions of the final manuscript. All authors read and approved the final manuscript.

## Author’s information

HMM is an Assistant Professor in the Department of Health Services Management and Policy at East Tennessee State University in the U.S., where he does research in tobacco use and control with particular focus on the WHO Framework Convention on Tobacco Control and Africa. HMM is a co-author of the book *Global Tobacco Control: Power, Policy, Governance and Transfer*. SPV is a postdoctoral research fellow in the Division of General Pediatrics, Department of Pediatrics at Vanderbilt University School of Medicine in the U.S., where he does research on pediatric respiratory outcomes in relation to several social and environmental risk factors, including tobacco. RMJ is an Assistant professor at the Indian Institute of Technology, Jodhpur, India. His research interests are primarily in the areas of health policy and lifestyle behaviors in the context of low- and middle- income countries. AEOO works in the WHO regional office for Africa and is the regional advisor for tobacco control. He has extensive experience in working with governments worldwide, particularly those in Africa.

## Pre-publication history

The pre-publication history for this paper can be accessed here:

http://www.biomedcentral.com/1471-2458/13/856/prepub
